# MELD Score Is Not Related to Spontaneous Bacterial Peritonitis

**DOI:** 10.1155/2015/270456

**Published:** 2015-07-01

**Authors:** Luciana Haddad, Tatiana Morgado Conte, Liliana Ducatti, Lucas Nacif, Luiz Augusto Carneiro D'Albuquerque, Wellington Andraus

**Affiliations:** ^1^Liver and Gastrointestinal Transplant Division, Department of Gastroenterology, University of São Paulo School of Medicine, Rua Aracaju 42, Ap. 41, 01240030 São Paulo, SP, Brazil; ^2^School of Nursing, University of São Paulo, Avenida Dr. Eneas de Carvalho Aguiar 419, 05403-000 Sao Paulo, SP, Brazil

## Abstract

This study investigates the correlation between SBP and repeated paracentesis, and its relation to MELD score, in cirrhotic patients with refractory ascites in an outpatient setting. Through the data base, 148 cirrhotic patients were prospectively included in the study with refractory ascites undergoing relief paracentesis from March 2012 to March 2013. Demographics data, etiology of liver disease, MELD score, and inscription on the waiting list for liver transplantation were analyzed. The ascites removed was analyzed through cellular count and culture for the diagnosis of spontaneous bacterial peritonitis. The cirrhotic patients underwent a total of 854 paracentesis procedures in the ambulatory setting during the study period. Eighty-one patients (54%) were on the waiting list for liver transplantation. Patients on the liver transplant list had higher associated costs due to a higher total number of outpatient paracentesis procedures (394.7 ± 512.3 versus 291.7 ± 384.7) and a higher volume drained per procedure (6.5 ± 8.5 versus 4.8 ± 6.4). There were 28 episodes of SBP (3.3%) diagnosed in 24 patients. In conclusion, the prevalence of asymptomatic SBP in cirrhotic patients with refractory ascites undergoing repeated paracentesis is low. MELD score is not related to spontaneous bacterial peritonitis.

## 1. Introduction

Ascites is the most common complication of cirrhosis [1–3]. Large-volume paracentesis performed on an outpatient basis is recommended for cirrhotic patients with ascites refractory to dietary and drug treatment [1–3].

Cirrhotic patients are more likely to develop bacterial infections and spontaneous bacterial peritonitis (SBP), which is the most frequent and potentially lethal complication of ascites [1–3]. The prevalence of SBP in cirrhotic patients is 10–30% in hospitalized patients [1, 3, 4] and has a yearly recurrence rate close to 70% [5]. SBP is associated with high mortality and the probability of long-term survival is 25–50% [6]. This infection has a poor prognosis [1, 2, 7] due to several severe complications, such as septic shock, progressive renal failure, variceal bleeding, and multiple organ failure [1, 2, 7].

Many factors are considered predisposing to the development of bacterial peritonitis in ascitic fluid. These risk conditions may be related to immunosuppression of the individual, increased intestinal permeability, bacterial growth in the small intestine [8], and an increase in bacterial translocation [9]. The correct and early identification of these conditions allows the clinical management appropriate to the patient [9], reducing the risk of complications associated with the development of SBP, such as portal hypertension, hepatorenal syndrome, and hyperdynamic circulation [8].

Approximately two-thirds of SBP cases are asymptomatic [1]; thus the protocols of clinical practice set forth by the American Association for the Study of Liver Disease and EASL recommend performing analysis of ascites fluid for diagnosis of infection after each paracentesis procedure [7]. However, this approach is debatable and there are few studies that evaluate the analysis of ascitic fluid in asymptomatic patients [5, 6].

The aim of this study was to investigate the correlation between SBP and repeated paracentesis, and its relation to MELD score, in cirrhotic patients with refractory ascites in an outpatient setting.

## 2. Methods

A number of 148 cirrhotic patients with refractory ascites who underwent paracentesis were followed up prospectively between the dates of March 2012 and March 2013 in the Liver and Gastrointestinal Transplant Division, Department of Gastroenterology, University of São Paulo School of Medicine (HCFMUSP), Brazil. The study included adults aged greater than 18 years who underwent outpatient paracentesis performed during the study period. The exclusion criteria were patients that did not want to participate in the study.

In Brazil, the MELD score is used as a criterion for allocating liver transplantation. It is also reported as marker of prognosis of some complications of liver cirrhosis, such as gastrointestinal bleeding and SBP. It is known that the higher the MELD, the greater the severity of liver disease and, therefore, the greater the need of the individual undergoing liver transplantation.

The MELD score was established after the determination of the calculation basis of this score as follows: INR, total bilirubin, creatinine, and sodium. After that the patients were divided into two groups for comparison of SBP development: patients in liver transplant waiting list and patients not listed for liver transplant. The patients listed for liver transplantation are those with MELD scores greater than or equal to 15 without contraindications, such as hepatobiliary metastasis or other organs; advanced cardiopulmonary disease; extrahepatic active infection; active alcoholism; drug abuse; being HIV positive; being positive for replication of hepatitis B virus; advanced chronic kidney disease.

Therapeutic paracentesis relief is indicated in patients with respiratory distress from tense ascites.

### 2.1. Description of the Procedure

Paracentesis procedures are performed Monday through Friday from 7:00 to 16:00 at the HCFMUSP Clinic for Surgery and Liver Transplantation. Local anesthetic with lidocaine chloridrate 2% (Xylocaine) is used prior to paracentesis. The puncture is performed in the left iliac fossa or the suprapubic midline using a 14-gauge Jelco IV catheter. Volume replacement is accomplished with 20% human albumin 50 mL when greater than 2 liters of ascites volume is removed and 1 vial (50 mL) of albumin is replaced for every 2 liters of ascites fluid removed. Routine analysis of ascites fluid cytology for diagnosis of possible asymptomatic SBP is performed on all clinical samples. Only after positive results or clinical suspicion (presence of fever, abdominal pain, and worsening of renal function) liquid cultures are performed.

Ascites fluid is collected in sterile bottles. The sample is centrifuged for 10 minutes and subjected to Giemsa staining techniques. Counts for the total number of polymorphonuclear cells are made by light microscopy. For bacterial culture, ascites fluid is inoculated into two blood culture bottles (each 10 mL) for aerobic and anaerobic analysis.

The diagnosis of SBP was established when the number of polymorphonuclear cells (PMN) was greater than or equal to 250 cells/mm^3^ regardless of the positivity of the bacterial culture [6]. The immunosuppression of the patient, increased intestinal permeability, bacterial growth in the small intestine, and increase in bacterial translocation were considered as risk factors for development of SBP.

This study was approved by the Ethics Committee of the Hospital das Clinicas, University of Sao Paulo, Sao Paulo, Brazil. Monetary values are expressed in US dollars. The values have been converted from Real to the Dollar according to the exchange in October, 2013, R $1 = US $$2.2.

### 2.2. Statistical Analysis

The results are described with mean and standard deviation. Median and range were used for currency values. Statistical analysis was performed using IBM-SPSS software version 20.0. Results were considered statistically significant with the *p* value being <0.05.

## 3. Results

A total of 148 patients with cirrhosis underwent a total of 854 paracentesis procedures in the ambulatory setting during the study period. The average patient age was 57 ± 11 years with a gender breakdown of 90 men (60.8%) and 58 women (39.2%). The average MELD score (Model for End-Stage Liver Disease) was 19.9 ± 8.45 (Table 1).

Patients underwent an average of 5.8 ± 7.8 paracentesis procedures/year. The total volume drained during one year was 7270.9 liters of ascites fluid and the average volume per patient was 8.4 liters. Fifty-two patients (35.1%) underwent only one paracentesis in the study period. During the study period, 28 (16%) died. In the patient population, the most common causes of cirrhosis were the following: viral hepatitis (B or C) (27%), alcohol cirrhosis (22.3%), and other causes (22.3%) (Table 1).

We divided the patients into two groups. Eighty-one patients (54%) were on the waiting list for liver transplantation during the study period, which included 51 men (63%) and 29 women (37%), with average age 55.9 years ± 11.1. Thirty patients died (20.3%) in the period, 26 on the waiting list for liver transplantation and 4 outside of it. In both lists, there was predominance of male deaths (76.7%). This difference was not statistically significant (*p* = 0.075).

There was no statistically significant difference between patients on and off the liver transplant list regarding age (55.9 ± 11.1 versus 59.4 ± 10.8) or the number of paracenteses performed (6.5 ± 8.5 versus 4.8 ± 6.4). Patients on the liver transplant list had higher associated costs due to a higher total number of outpatient paracentesis procedures ($391.7 range $210.3–$680.8 versus $282.7 range $156.6–$545.6), a higher volume drained per procedure (6.5 ± 8.5 versus 4.8 ± 6.4), and higher MELD score (22.9 ± 8.7 versus 14.8 ± 5.9). Additionally, there were more deaths during the study period in the patient group on the liver transplant list (30% versus 5.7%) compared to patients not on the transplant list (*p* < 0.001) (Table 2). The patients on the transplant waiting list had less SBP (11.3%) than those not listed (22.1%), although it does not reach statistical significance (*p* = 0.075) (Table 2).

There were 28 episodes of SBP (3.3%) diagnosed in 24 patients. Of these, 24 (85.7%) were asymptomatic SBP, while 4 were symptomatic, presenting one or more symptoms, such as fever, abdominal pain, hepatic encephalopathy, recent gastrointestinal bleeding, or worsening renal function.

The total number of patients was 192, with 58% of men and 42% of women. They underwent an average of 8 paracenteses per patient, counting a total of 854 paracenteses (Table 3). Among 24 patients that had SBP, only eight (33.3%) were in liver transplant waiting list. Patients with SBP in the transplant list had a higher volume drained per procedure (66.4 ± 63.4 versus 49.2 ± 10.6; *p* = 0.012) and higher MELD score (20.7 ± 7.1 versus 13.3 ± 4.9; *p* < 0.001), although the SBP rate was not different (Table 4).

## 4. Discussion

The prevalence of SBP in cirrhotic outpatients undergoing large volume paracentesis is low, which is confirmed by this study (Table 5). Mohan and Venkataraman [1] conducted a prospective study from January 2008 to December 2009 with 110 cirrhotic patients who underwent a total of 278 outpatient paracenteses. During this period, that study found 7 cases of asymptomatic SBP (2.5%) and 3 cases with positive bacterial culture results. Their results are similar to ours, as they also showed that the diagnosis of asymptomatic SBP has a low incidence.

To our knowledge, this is the study with the biggest number of paracenteses per patient reported in the literature (Table 4). This fact confirms the severity of the refractory ascites and the liver disease on these patients. However, despite the big number of paracenteses and the amount of liquid removed, the rate of SBP was low (3.3%), and it is in accordance with the literature (0.5–3.5%). One reason for the low rate of SBP is the fact that we have a dedicated facility and human resources for cirrhotic patients procedures, like liver biopsies and paracentesis.

According to a retrospective study by Evans et al. [11], the prevalence of SBP was 3.5% of 427 cases in cirrhotic outpatients, which included 6 (1.4%) and 9 patients with positive and negative (2.1%) culture results, respectively.

In another study [12] involving 1041 patients with cirrhosis and 355 outpatients with ascites, a total of 2123 paracenteses were performed. In ambulatory patients, the diagnosis of SBP occurred in only 3.1% of cases and 1.2% of SBP cases were asymptomatic. Castellote et al. [10] obtained a rate of asymptomatic SPB of only 0.5% in cirrhotic patients who underwent a total of 204 outpatient paracentesis procedures.

Despite the low rate of SBP in asymptomatic cirrhotic patients, the analysis of the removed ascites is necessary because it is the only way to diagnose SBP in these cases. In addition, the treatment in the early phase of infected ascites can prevent patient worsening, hospitalization, and death, since those with refractory ascites are already very ill patients.

The patients in the transplant waiting list presented the biggest amount of drained ascites, leading to a higher cost of the procedure, and also an increased amount of albumin replacement. In addition, despite the fact that they had less SBP, their mortality rate was higher, explained by the disease severity and the higher MELD score. Currently, worldwide there was a decrease in the general incidence of SBP in cirrhotic patients because those with worse liver function are undergoing liver transplantation. However, in regions where there is still a big transplant waiting list, there are for sure many patients with refractory ascites. It is well known that mortality is related to the MELD score, and as demonstrated here patients with higher MELD score have higher mortality rates. However, paracentesis has been shown to be a safe procedure, and it was not related to death. Repeated paracentesis is still a good therapeutic option for refractory ascites.

Patients in the transplant waiting list presented less SBP and they had a significant higher MELD score (23 versus 15). Thus, the development of SBP is not related to the MELD score, but instead it is related to the clinical status of the refractory ascites and the need of repeated paracentesis.

## 5. Conclusion

The prevalence of SBP in asymptomatic cirrhotic patients is low. Our study showed that the occurrence of SBP is not related to the severity of liver disease evaluated by MELD score. Repeated paracentesis is still a good palliation for refractory ascites until the definitive treatment with liver transplant.

## Figures and Tables

**Table 1:** Demographic and clinical characteristics of 148 patients who underwent paracentesis relief.

Male, *n* (%)	90 (60.81)
Age (years), mean ± SD	57 ± 11
Amount of paracentesis, mean ± SD	5.8 (1–47)
Amount of drained volume (liters), mean ± SD	8.4 (0–20)
MELD score, mean ± SD	19.9 ± 8.45
Cirrhosis' etiology, *n* (%)	
Alcohol	33 (22.3)
Viral hepatitis (B ou C)	40 (27)
Others	33 (22.3)
Deaths, *n* (%)	
Male	22 (14.8)
Female	6 (4)

**Table 2:** Comparison between patients listed for liver transplantation and other patients.

Variable	Patients listed (*n* = 80)	Other patients (*n* = 68)	*p*
Gender, *n* (%)			
Male	51 (63.8%)	39 (57.4%)	0.42
Female	29 (36.2%)	26 (42.6%)	
Liters per patient, mean ± SD	55.9 ± 11.1	59.4 ± 10.8	0.06
Paracentesis per patient, mean ± SD	6.5 ± 8.5	4.8 ± 6.4	0.17
MELD score, mean ± SD	22.9 ± 8.7	14.8 ± 5.9	<0.001
Cost of procedure ($), mean ± SD	394.7 ± 512.3	291.7 ± 384.7	0.04
Death, *n* (%)	24 (30%)	4 (5.7%)	<0.001
SBP, *n* (%)	9 (11.3%)	15 (22.1%)	0.075

SBP: spontaneous bacterial peritonitis.

**Table 3:** Characteristics of patients with spontaneous bacterial peritonitis undergoing large volume paracentesis.

Series	Age (years)	Sex	Etiology of cirrhosis	Aspect of ascitic fluid	Polymorphonuclear cells/mm^3^ of ascitic fluid	Organism in culture	MELD	Death
1	67	M	Alcohol	Turbid	2790	Negative	18	Yes
2	69	M	Hepatitis B virus	Limpid	1023	No culture	23	Yes
3	56	M	Undiagnosed	Hemorrhagic	446.5	No culture	7	No
4	58	M	Undiagnosed	Turbid	5715	No culture	9	No
5	65	M	Undiagnosed	Turbid	38115	Negative	17	No
6	65	M	Undiagnosed	Limpid	52136	No culture	10	No
7	70	F	Cryptogenic	Limpid	1334.8	Negative	20	No
8	67	M	Undiagnosed	Slightly turbid	850.5	No culture	13	No
9	60	F	Cryptogenic	Turbid yellow	6935.5	*Streptococcus viridans *	22	No
10	58	M	HCC	Hemorrhagic	307.8	No culture	7	No
11	64	M	Alcohol + HCC	Slightly turbid	262.2	No culture	13	No
12	64	F	Cryptogenic + schistosomiasis	Limpid	387.5	No culture	10	No
13	64	F	Cryptogenic	Limpid	2044.8	No culture	13	Yes
14	59	M	Hepatitis B virus	Turbid	273.25	Negative	17	No
15	54	F	Undiagnosed	Limpid	270	No culture	14	No
16	57	F	Hepatitis C virus	Slightly turbid yellow	4606	Negative	14	No
17	52	F	Schistosomiasis	Limpid	314	No culture	11	No
18	52	F	Undiagnosed	Limpid	280	No culture	10	Yes
19	52	F	Schistosomiasis	Limpid	360	No culture	13	No
20	50	F	HAI	Turbid yellow	1822.5	Negative	14	No
21	65	M	Alcohol	Turbid	1740	*Staphylococcus capitis *	13	No
22	55	M	Alcohol	Limpid	422.4	No culture	36	Yes
23	52	M	Hepatitis C virus	Turbid	294	No culture	29	Yes
24	63	M	Undiagnosed	Slightly turbid yellow	258.5	No culture	12	Yes
25	69	M	Undiagnosed	Slightly turbid	317.25	Negative	16	Yes
26	52	F	Hepatitis C virus	Turbid yellow	2527.2	No culture	14	Yes
27	62	F	Hepatitis B virus	Limpid	3741	No culture	22	No
28	70	M	Hepatitis C virus	Turbid	5161.5	No culture	16	No

**Table 4:** Comparison of spontaneous bacterial peritonitis in cirrhotic patients list and not list for liver transplant.

Variable	Waiting list (*n* = 8)	Not waiting list (*n* = 16)	*p*
Gender, *n* (%)			
Male	4 (16.6)	12 (50.0)	0.63
Amount of paracentesis, mean	8.7 ± 6.8	6.8 ± 9.6	0.56
Amount of drained volume (liters), mean	66.4 ± 63.4	49.2 ± 10.6	0.012
MELD score, mean ± SD	20.7 ± 7.1	13.3 ± 4.9	<0.001

**Table 5:** Prevalence of spontaneous bacterial peritonitis in cirrhotic patients undergoing large volume paracentesis in outpatient clinic.

Reference	Number of patients	Number of paracenteses	Prevalence of PBE
Mohan and Venkataraman [1]	110	278	7 (2.5%)
Castellote et al. [10]	40	204	1 (0.5%)
Evans et al. [11]	427	427	15 (3.5%)
Cadranel et al. [12]	355	976	65 (1.2%)
Present study	148	854	24 (3.3%)
